# Spectral Effects on* Symbiodinium* Photobiology Studied with a Programmable Light Engine

**DOI:** 10.1371/journal.pone.0112809

**Published:** 2014-11-12

**Authors:** Daniel Wangpraseurt, Bojan Tamburic, Milán Szabó, David Suggett, Peter J. Ralph, Michael Kühl

**Affiliations:** 1 Plant Functional Biology and Climate Change Cluster, University of Technology Sydney, Sydney, Australia; 2 Marine Biological Section, Department of Biology, University of Copenhagen, Helsingør, Denmark; 3 Singapore Centre on Environmental Life Sciences Engineering, School of Biological Sciences, Nanyang Technological University, Singapore, Singapore; University of Vigo, Spain

## Abstract

The spectral light field of *Symbiodinium* within the tissue of the coral animal host can deviate strongly from the ambient light field on a coral reef and that of artificial light sources used in lab studies on coral photobiology. Here, we used a novel approach involving light microsensor measurements and a programmable light engine to reconstruct the spectral light field that *Symbiodinium* is exposed to inside the coral host and the light field of a conventional halogen lamp in a comparative study of *Symbiodinium* photobiology. We found that extracellular gross photosynthetic O_2_ evolution was unchanged under different spectral illumination, while the more red-weighted halogen lamp spectrum decreased PSII electron transport rates and there was a trend towards increased light-enhanced dark respiration rates under excess irradiance. The approach provided here allows for reconstructing and comparing intra-tissue coral light fields and other complex spectral compositions of incident irradiance. This novel combination of sensor technologies provides a framework to studying the influence of macro- and microscale optics on *Symbiodinium* photobiology with unprecedented spectral resolution.

## Introduction

Scleractinic corals form the basis of one of the most productive and biodiverse ecosystems on Earth, coral reefs. The success of corals in tropical, nutrient poor waters is based on the symbiotic interaction between the coral cnidarian host and its microalgal endosymbionts, i.e., dinoflagellates belonging to the genus *Symbiodinium*. Microalgal photosynthesis generates O_2_ and photosynthates in the form of simple carbohydrates, to fuel the host metabolism, whilst in return the host provides a protective environment and nutritious metabolic waste products that sustain photosynthesis by the algae [1].

The light-dependency of *Symbiodinium* photosynthesis, physiology and growth has been studied in detail, involving mainly fluorescence-based and O_2_-evolution based approaches [2]–[4]. Variable chlorophyll (Chl) *a* fluorescence is frequently used to estimate *Symbiodinium* light use efficiency and photoinhibition (i.e. the decrease in photosynthetic quantum efficiencies under excess irradiance). *Symbiodinium* can downregulate photosystem II (PSII) activity to protect against excess irradiance [5]; this is achieved largely via non-photochemical quenching (NPQ), mediated by the xanthophyll cycle [6] and enhanced energy dissipation of the light harvesting antenna complexes [7]–[8]. O_2_-evolution based approaches (e.g. using O_2_ optodes) usually measure net O_2_ evolution, that together with an estimate of light respiration approximates gross photosynthesis [9]. In light, O_2_ consumption by the algae is fuelled by photosynthesis and thus light respiration is enhanced over dark respiration [10]–[12]. Upon darkening of an illuminated *Symbiodinium* sample, respiration is enhanced for some time (several minutes), this period is known as light-enhanced dark respiration (LEDR) and it is frequently used as a proxy for light respiration.

Studies on coral photobiology have largely been performed on *Symbiodinium* cultures with artificial light sources under controlled conditions [13]–[15]. In most cases, such investigations involve measurements of the integrated photon irradiance of photosynthetically available radiation (PAR; 400–700 nm), while the spectral composition of PAR is not considered [15]. However, light can have wavelength-specific effects on coral photosynthesis [16]–[17], mainly due to the distinct action spectrum of *Symbiodinium* photosynthesis, showing highest efficiency in blue light [15]–[16], [18]–[19]. Given the frequent use of artificial light sources in laboratory studies on coral photosynthesis and bleaching, it is thus important to test whether spectral modifications due to the use of artificial light sources affect S*ymbiodinium* photophysiology and therefore limit our ability to extrapolate laboratory-based photosynthesis studies to the microenvironmental conditions that occur *in hospite* [20]–[22].

The spectral composition of artificial light sources in laboratory-based studies of *Symbiodinium* is often red-shifted relative to natural sunlight, as cool-white fluorescent lamps [2], [23] and halogen light sources [21], [24]–[26] are commonly used. However, the spectral light field that *Symbiodinium* receives in nature is modulated on many different spatial and temporal scales and can be very different from such red-shifted white-light spectra [21], [27]–[29]. Upon entering the water column, the spectral composition of sunlight changes rapidly due to the absorption in the red-infrared spectral region by pure water [30]; additional contributions to spectral shifts by dissolved and particulate components are usually insignificant for the oligotrophic waters of most reefs [31]. As solar irradiance is attenuated exponentially with increasing water depth, the spectral composition of light is continuously shifting towards enrichment in shorter wavelengths, and corals located in deeper waters (>30 m) are thus primarily exposed to blue light [27].

The variability of coral reef light environments becomes more complex when considering the microscale [29], and thus the spectral light field experienced by individual *Symbiodinum* cells within the animal tissue. Recent studies on the optical properties of coral tissue and skeleton have revealed that vertical light attenuation is predominately in the blue region, thus leaving more red light available for symbionts in deeper tissue layers [21]. Corals also harbor various types of fluorescent and chromophoric host pigments that absorb energy-rich UV, blue and blue-green light and cause red shifted reemission [26], [32]. The optical properties of corals thus strongly determine the actual *in hospite* light regime for *Symbiodinium* photosynthesis.

In this study, we used information from microscale light measurements in coral tissue in combination with a programmable light engine to reconstruct the intra-tissue spectral irradiance of a coral in lab studies of *Symbiodinium* photosynthesis and respiration, and we compared such measurements with data obtained with a halogen lamp spectrum. Specifically, we determined O_2_ evolution and LEDR of *Symbiodinium* using O_2_ optodes as well as PSII electron transport rates based on variable Chl *a* fluorescence measurements. Our study provides new approaches to studying coral photobiology and we discuss our findings with a focus on a basic understanding of spectral dependence of *Symbiodinium* photosynthesis and respiration.

## Methods

### 2.1. *Symbiodinium* culture

*Symbiodinium* strain CS-73 (clade A) originally isolated from Heron Island, in the southern Great Barrier Reef of Australia (Australian National Algae Culture Collection, Commonwealth Scientific and Industrial Research Organisation; www.csiro.au) was cultured in f/2 medium prepared with artificial seawater [33]. Algae were cultured in round-bottom flasks, wherein the medium was flushed with air through a sterile glass pipette to enhance growth and prevent clumping [34]–[35]. The culture was grown at constant temperature (25°C) and salinity (33) under white fluorescent light tubes and incident downwelling irradiance (400–700 nm; 12/12 h light-dark cycle) of ∼50 µmol photons m^−2^ s^−1^.

To minimise effects of culture aging on *Symbiodinium* photosynthesis, we only used cells that were in exponential growth phase (between 13–19 days relative to start of culture), as determined by daily cell counts using a cell counter (Beckman Coulter GmbH, Krefeld, Germany). Our different spectral treatments were assigned randomly across different experimental days to integrate potential ‘time of day effects’ since preliminary experiments demonstrated that *Symbiodinium* photosynthesis was affected by the time of the day (morning vs. mid-day/afternoon). This was likely related to observed differences in the ambient O_2_ concentration of the culture and differences in the illumination history during the morning and mid-day measurement periods [36]–[37]. However, to overcome such issues, a small amount of culture, i.e., the measuring volume of 1.6 mL was maintained dark (wrapped in aluminum foil) in the growth incubator for a period of 12 h. Following this period, the ambient O_2_ concentration was brought to air saturation by flushing the culture with air through a pipette tip. In this way, we minimized the time-of-day effect on photosynthesis but also provided reoxygenation of the cell suspension, which is required to eliminate O_2_ depletion due to respiration during the dark-adaptation period. This procedure was therefore repeated prior to all measurements.

All experiments were standardized to a similar cell density [34]. Briefly, 1 mL of culture was centrifuged (5 min at 1,550 g), fixed with 4% formaldehyde and stored at 4°C. Prior to each measurement, the samples were centrifuged, washed and resuspended in 100% (4°C) chilled methanol, then sonicated (3×1 min) in an ice-water bath. This procedure breaks up cell aggregates without breaking the cells themselves [34]. Finally, cell density was measured using a cell counter (as above). The cell densities in the aliquots for experimentation were then adjusted using fresh media to final cell concentrations of between 43,300 and 70,200 cells mL^−1^ ( = F_0_∼1 at a MC-PAM measuring light wavelength of 440 nm).

### 2.2. Actinic light spectra

**Figure 1:**
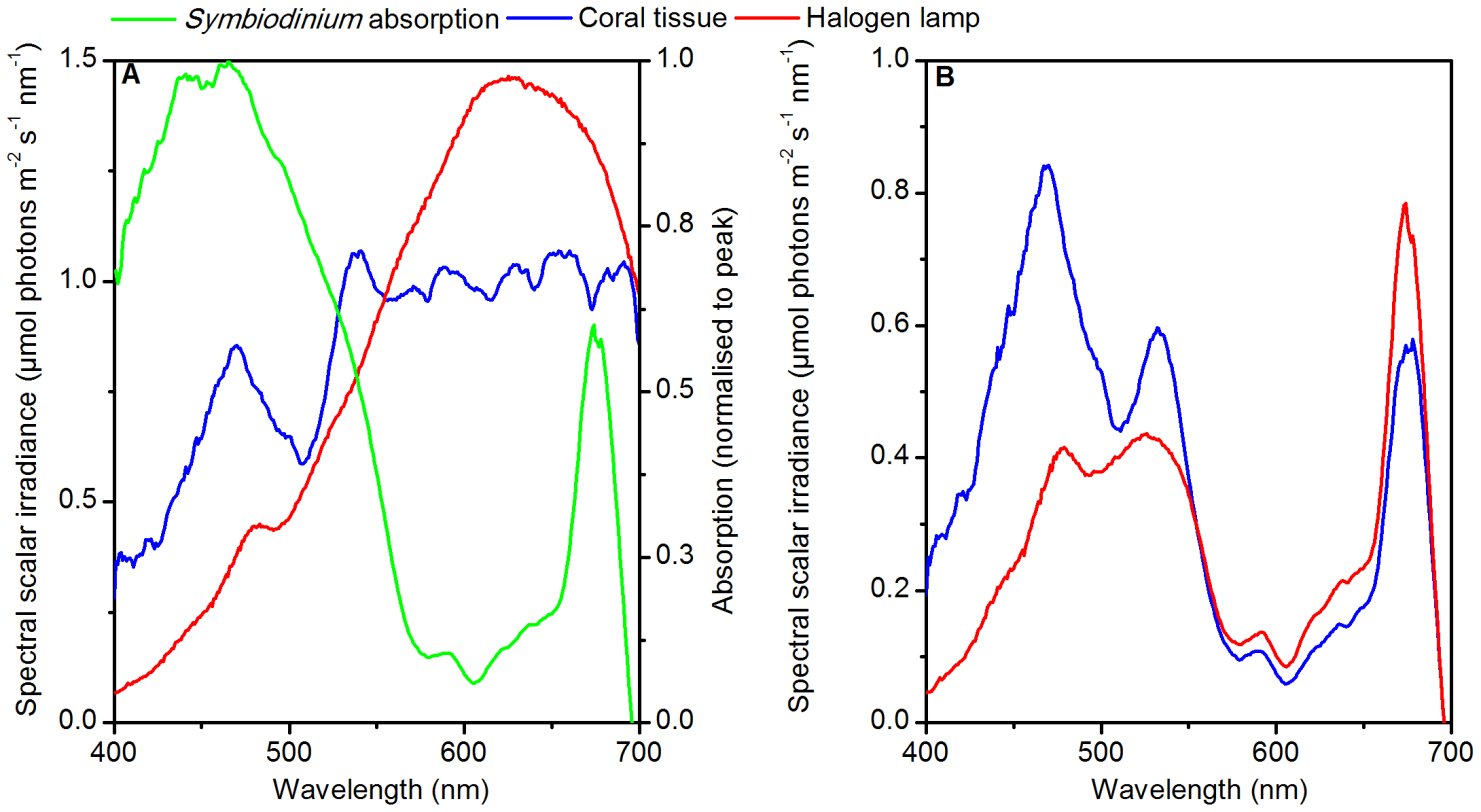
Light spectra and *Symbiodinium* absorption. A) Normalised absorption spectrum of *Symbiodinium*, and defined scalar irradiance spectra used in the experiments in which the OL 490 light engine was used to simulate the *in situ* light exposure (‘coral tissue’, blue) and ‘halogen lamp’ (red) exposure (in µmol photons m^−2^ s^−1^ nm^−1^). The scalar irradiance spectra were normalised to give the same integrated output in µmol photons m^−2^ s^−1^ over PAR (400–700 nm). The absorption spectrum of *Symbiodinium* was normalised to its peak absorption at 465 nm. B) Photosynthetically usable radiation (PUR; see text).

We applied two spectral irradiance regimes to *Symbiodinium* simulating: a) the *in situ* light spectrum within the tissue of the common coral *Favites abdita* (‘coral tissue’, Fig. 1A), and b) a white light spectrum as provided by a fiber-optic tungsten-halogen lamp (KL-2500, Schott, Germany) typically used in laboratory experiments (‘light source’, Fig. 1A). For the coral tissue spectrum, we first measured the incident scalar irradiance spectrum on a shallow coral reef flat (Heron Island lagoon, Great Barrier Reef) at ∼0.7 m depth during solar noon with a custom-made fiber-optic spectrometer connected to a scalar irradiance sensor [29]. We also estimated the spectral distribution of light received within the coral tissue using a fragment of *Favites abdita* under laboratory conditions [21]. Here, we used scalar irradiance microsensors [21], [38] that were positioned within the upper 100 µm of coral tissue, which is the tissue depth where *Symbiodinium* is likely to occur. The spectral counts between 400–700 nm were normalised to the incident downwelling irradiance obtained under experimental conditions (Fig. S1, [21]). Spectral irradiance experienced by *Symbiodinium* within the tissue under *in situ* conditions was then estimated by multiplying this normalized coral tissue spectrum under experimental conditions with the incident *in situ* spectrum obtained on a shallow water coral reef.

A novel light engine was used (OL 490 Agile Light Source, Gooch & Housego, Orlando, Florida, USA) to reproduce the spectrally defined actinic irradiance for the *Symbiodinium* light exposure experiments. Usually, narrow-bandwidth spectra are generated using diffraction gratings, filters or monochromators, which produce a single bandwidth of light and thus require to be tuned to subsequent wavelengths. In contrast, the OL 490 uses an advanced digital light processing microchip (Texas Instruments, Dallas, Texas, USA) to produce freely defined complete spectra with a specified irradiance and spectral resolution. In our configuration, the OL 490 could deliver a photon irradiance of 1,230 µmol photons m^−2^ s^−1^ onto a 1 cm^2^ surface at a spectral bandwidth precision of ±2 nm.

The spectral photon irradiance of the two spectra (‘coral tissue’ and ‘halogen lamp’) generated by the OL-490 (µmol photons m^−2^ s^−1^ nm^−1^) was adjusted so that both spectral regimes exhibited an identical photon irradiance integrated over PAR (400–700 nm; Fig. 1A) for each irradiance level (47, 59, 130, 213, 336, 417, 719, and 999 µmol photons m^−2^ s^−1^). The spectra were interpolated to bandwidths of 0.3 nm; in this format, the spectra could be entered into the OL 490 light engine to generate a semi-continuous spectrum. Photosynthetically usable radiation was calculated (PUR; Fig. 1B,) for *Symbiodinium* as [39]:
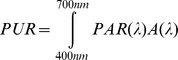
where *PAR*(λ) denotes the incident spectral irradiance, and *A*(λ) is a weighted probability function that a photon will be absorbed by *Symbiodinium* at a given wavelength, λ. We calculated *A*(λ) by normalizing the *Symbiodinium* absorption at a given wavelength to its absorption maximum [39].

### 2.3. Experimental setup and procedure

**Figure 2:**
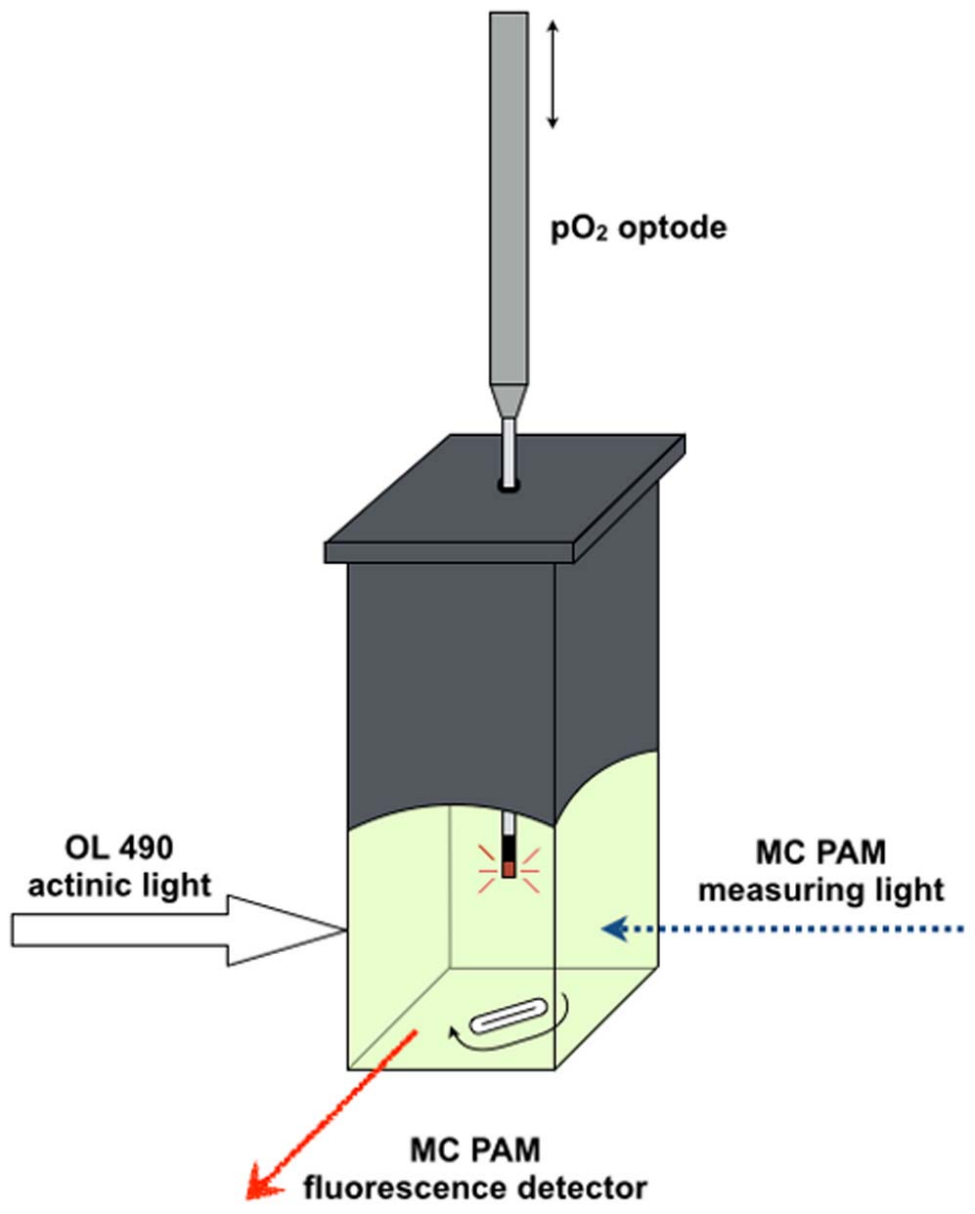
Cuvette-based set-up for combined O_2_ exchange and variable chlorophyll fluorescence measurements on *Symbiodinium* cultures. The O_2_ optode was inserted into the cuvette through a small hole in the gas-tight lid. Actinic light was provided by the OL-490 light engine equipped with a liquid light guide and collimator. The MC-PAM provided measuring light in opposite position to the actinic light and at right angles to the fluorescence detector. The cuvette temperature was controlled at 25°C by an ambient water bath and heater.

A cuvette-based incubation system was used to monitor the physiological response of *Symbiodinium* to different spectral light exposure (Fig. 2). *Symbiodinium* samples were incubated in a 1 cm rectangular quartz cuvette with a custom-designed gas seal and a working volume of 1.6 mL. One side of the cuvette was illuminated with actinic light generated by the OL 490 light engine (see above).

The photophysiological response of *Symbiodinium* to different spectral treatments was investigated using both O_2_ evolution and variable Chl *a* fluorescence measurements via optodes and pulse-amplitude modulation (PAM) fluorometry. Fluorescence was measured using a Multi-Colour PAM fluorometer (MC PAM; Heinz Walz GmbH, Effeltrich, Germany) [40]–[41]. The sample holder of the MC PAM controls the experimental temperature within the cuvette via its connection to a thermostatic water bath. The delivery of ‘measuring light’ for the MC-PAM and the fluorescence detector were located on adjacent cuvette-faces (Fig. 2). The culture was continuously stirred with a small magnetic stirrer bar in the cuvette.

The gas-tight lid of the cuvette was carefully closed ensuring the sample was free of air bubbles. The underside of the lid is concave so that air bubbles escape through a 1.2 mm diameter hole in the middle of the lid. A fiber optic O_2_ optode (PyroScience GmbH, Aachen, Germany) was inserted through the cuvette lid using a micromanipulator (MM33, Märtzhäuser GmbH, Germany); this optode was a fixed needle-type minisensor (1.1 mm tip diameter) with a black optical isolation and a 90% response time of <3 s, and calibrated in air-saturated water (100% air saturation) and O_2_-free seawater (flushed with N_2_) at the experimental temperature (25°C) and salinity (S = 33). The percent air saturation was transformed to µM oxygen as in Garcia and Gordon [42]. The optode was connected to an O_2_ data logger (FireSting, PyroScience, Fibreoptic Oxygen Meter FS02-01) and controlled by the manufacturer's software (PyroScience, FireSting Logger V 2.365) running on a PC that was interfaced to the O_2_ data logger via an USB interface. Dissolved O_2_ was measured continuously at a sampling interval of 1 s.

Initially, each *Symbiodinium* sample was dark-adapted for 30 min to estimate dark respiration. Each sample was then illuminated for 10 min followed by 10 min of darkness; this protocol was sufficient to ensure robust estimates of O_2_ production and consumption from linear changes in chamber O_2_ concentration over time (data not shown). Light-dark transitions were sequentially performed under increasing photon irradiance regimes of 47, 86, 130, 173, 213, 253, 336, 417, 719 and 999 µmol photons m^−2^ s^−1^ as provided by the programmed spectra, generated by the OL 490 light engine to yield the photosynthesis-light response relationship. PAM fluorometry was used simultaneously to measure the maximum quantum efficiency (*F_v_*/*F_m_*) of PSII and the operating efficiency of PSII (Φ_PSII_) under dark and light conditions, respectively. Blue pulse-amplitude-modulated measuring light (<0.5 µmol photons m^−2^ s^−1^, 440 nm) was generated by the PAM while actinic light was provided by the OL 490 light engine. Saturating pulses (2,000 µmol photons m^−2^ s^−1^, 440 nm, 0.8 s pulse width) were applied after the 30 min of dark acclimation and at the end of each illumination period. Relative electron transport rates (rETR) were calculated by multiplying Φ_PSII_ with the corresponding incident PAR irradiance [43] and converted to a proxy for absolute electron transport rates by multiplying Φ_PSII_ with PUR.

### 2.4. Data analysis

Changes in dissolved O_2_ concentration in the cuvette were used to calculate photosynthesis and respiration. The magnitude of LEDR is known to change in proportion to that of O_2_ consumption in the light [44]–[45], and has therefore been used as a proxy for light respiration [9], [12].

Net photosynthesis and LEDR were calculated based on the linear change in O_2_ concentration measured during each of the 10 min incubation periods [9]. The sum of net photosynthesis and LEDR was used as an estimate of extracellular gross O_2_ production [9]. This extracellular O_2_ production does not equal the intracellular formation of O_2_ and thus the rate of water splitting and the oxidation state of PSII. Our extracellular gross photosynthesis estimate has to be regarded as conservative, considering that true light respiration, i.e., the respiration that occurs during illumination, is likely to be higher than LEDR [11], [46]. Production and consumption rates of O_2_ for each experiment were normalized to the respective cell density.

LEDR was fitted to a first order exponential decay function. Gross photosynthesis (P_G_), relative and absolute ETR (rETR and ETR) curves versus irradiance were analysed according to the empirical equation of Platt et al. [47] which was applied as a fitting routine according to Ralph and Gademann [48]. The parameters obtained as a result of the fitting procedure were 1) *α*, photosynthetic rate in light-limited region of the light curve, 2) *E*_k_, minimum saturating irradiance and 3) ETR_max_ and P_max_, maximum electron transport rate and maximum photosynthesis rate, respectively. Statistical differences in photosynthetic performance of *Symbiodinium* (P_max_ or ETR_max_, α and E_k_) between “coral tissue” and “halogen lamp’ illumination were tested for by using a 2-tailed student's t-test (α level = 0.05).

## Results

### 3.1. Effects of spectral light composition on O_2_ turnover

**Figure 3:**
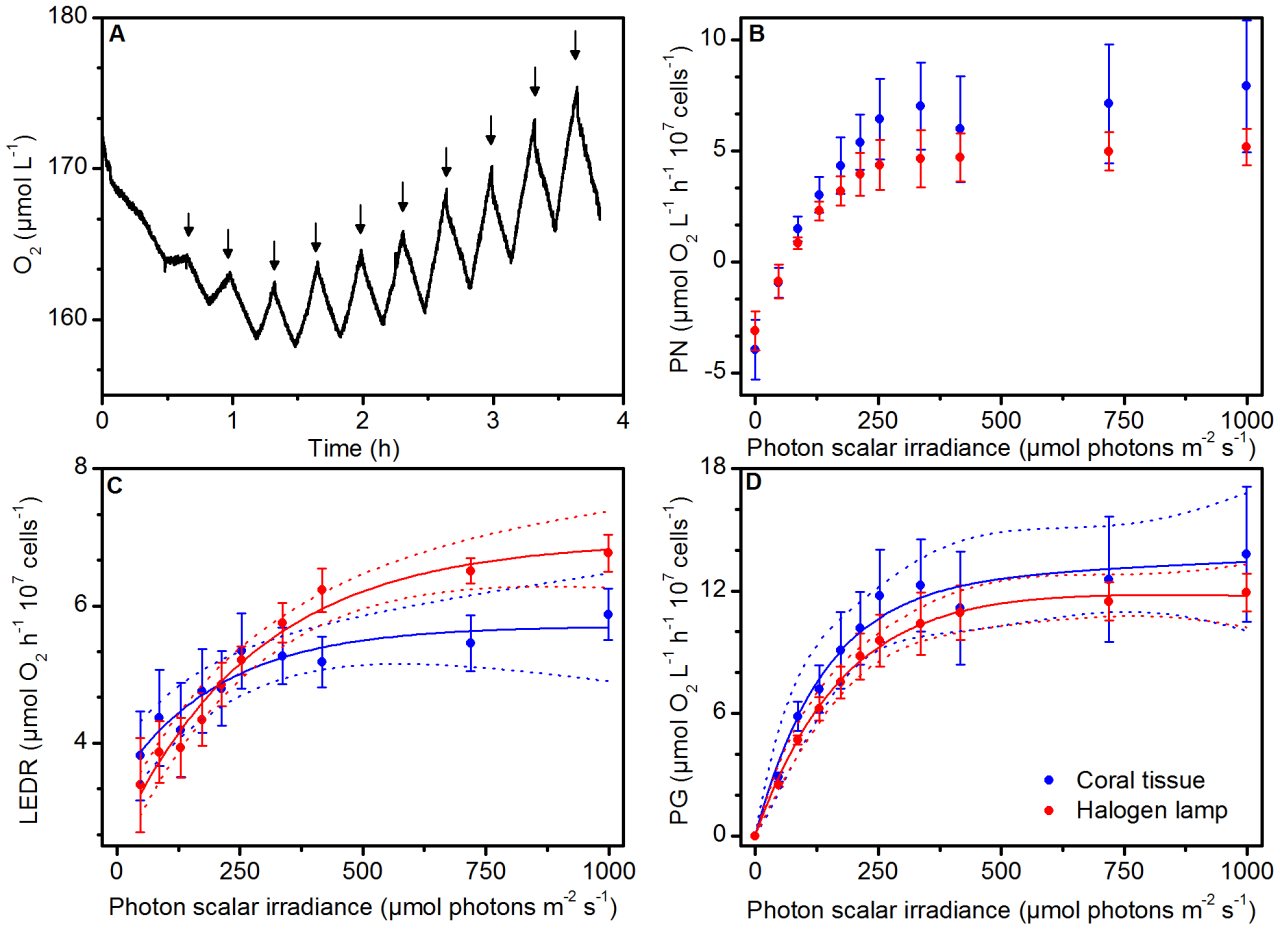
Effect of spectral light composition on *Symbiodinium* O_2_ turnover. (A) Example of O_2_ dynamics in a *Symbiodinium* culture sample under experimental light-dark transitions of increasing irradiance. Black arrows show the onset of a 10 min dark period followed by a 10 min illumination periods at progressively higher irradiances. The slopes during each period of illumination and darkness were used to calculate *Symbiodinium* net photosynthesis (panel B; PN) and light-enhanced dark respiration (panel C, LEDR), respectively. The sum of PN and LEDR estimated gross photosynthesis (panel D; PG). Experiments were done under 11 experimental irradiance regimes (including darkness). The photon scalar irradiance integrated over 400–700 nm was equal for the two spectral treatments (‘coral tissue’ and ‘halogen lamp’) at each respective irradiance level. Solid lines indicate best fits and dotted lines represent 95% confidence intervals. Symbols and error bars indicate the mean ± SE (*n* = 3).

Light-dark transitions led to linear changes in O_2_ concentration within the cuvette-system (Fig. 3A). Upon darkening, we observed an initial rapid phase of O_2_ depletion (<3 s) that quickly slowed down to a steady rate of O_2_ depletion over the 10 min dark phase. Net and gross photosynthesis of *Symbiodinium* showed similar changes with irradiance for the two spectral treatments (Fig. 3B, D). For instance, P_max_ was ∼13.5 and 12 µmol O_2_ h^−1^ 10^7^ cells^−1^ with a minimum saturating irradiance, E_k_, of 156 and 171 µmol photons m^−2^ s^−1^ for coral tissue (r^2^>0.84) and halogen lamp (r^2^ = 0.98), respectively (Fig. 3D, Table 1).

**Table 1:** Photosynthetic performance of *Symbiodinium* under defined broadband spectra simulating *in situ* coral tissue and halogen lamp spectral composition.

	PG		rETR		ETR	
	Tissue	Lamp	Tissue	Lamp	Tissue	Lamp
P_max_ or ETR_max_	13.49 (±3.11)	11.96 (±1.21)	109.3 (±7.5)	126.6 (±7.9)	47.5 (±3.3)	41.0 (±2.6)
α	0.09 (±0.02)	0.07 (±0.01)	0.81 (±0.01)	0.80 (±0.01)	0.35 (±0.01)*	0.26 (±0.003)*
E_k_	156.4 (±29.4)	170.7 (±14.3)	135.9 (±10.9)	157.9 (±9.4)	135.9 (±10.9)	157.9 (±9.4)

The photosynthetic parameters P_max_ (in O_2_ h^−1^ 10^7^ cells^−1^) or ETR_max_, α, and E_k_ (in µmol photons m^−2^ s^−1^) were derived from O_2_ evolution based (P_G_) and Chl *a* fluoremetry-based (rETR and ETR) photosynthesis irradiance curves. A significant difference (p<0.05) between coral tissue and halogen lamp is denoted with an asterisk. Means (± SE) are shown (*n = 3*).

LEDR increased with increasing photon irradiance and values at low light (i.e. 44 µmol photons m^−2^ s^−1^) increased by about 1.5 and 2 times under the highest irradiance (∼1000 µmol photons m^−2^ s^−1^) for the halogen lamp (r^2^ = 0.78) and coral tissue spectrum (r^2^ = 0.30), respectively (Fig. 3C). Above 300 µmol photons m^−2^ s^−1^, the red-weighted halogen lamp spectrum induced ∼15–20% higher LEDR rates than the coral tissue spectrum (Fig. 3C). For instance, at ∼400 µmol photons m^−2^ s^−1^, the mean O_2_ consumption (in µmol O_2_ h^−1^ 10^7^ cells^−1^ was 6.2 (±0.31 SE) and 5.2 (±0.37 SE) for the halogen lamp and coral tissue spectrum, respectively, but these differences were not statistically significant (2-tailed *t* test, t(2.2) = 4, p = 0.09).

### 3.2. Spectral light effect on variable Chl *a* fluorescence

**Figure 4:**
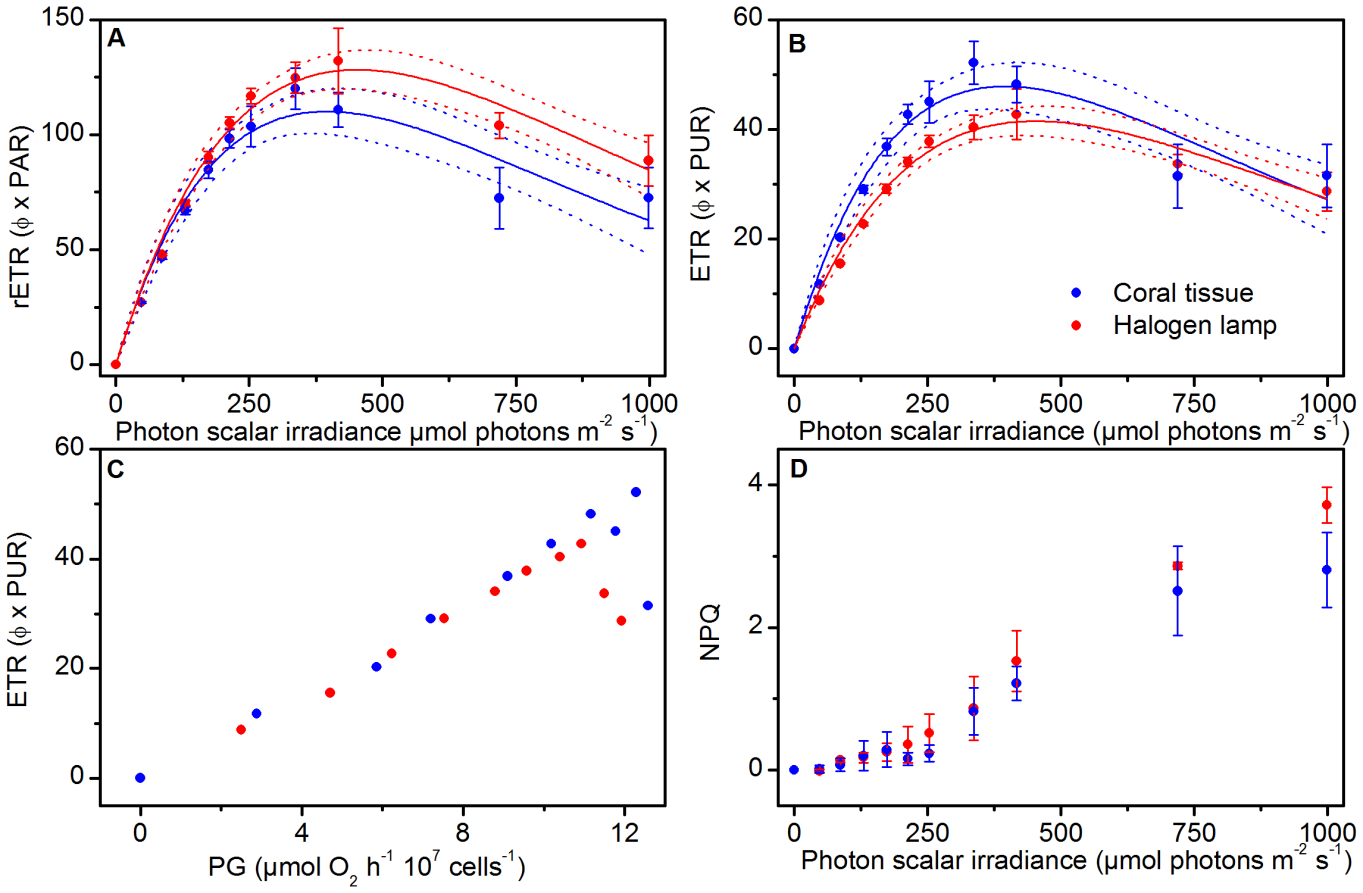
Effect of spectral light composition on (A) rETR vs. PAR and (B) ETR vs. PAR. (C) Relationship between gross photosynthesis (PG) and ETR. (D) Non-photochemical quenching (NPQ). Note that the y-axis scale in (A) and (B) differ. Solid lines indicate best fits and dotted lines represent 95% confidence intervals (r^2^>0.91). Symbols and error bars represent the mean ± SE (*n* = 3).

The rETR vs. irradiance curves were similar for the *in situ* coral tissue spectrum and halogen lamp spectrum under photon irradiances below ∼400 µmol photons m^−2^ s^−1^, which was about the irradiance where rETR saturated (Table 1, Fig. 4A). At irradiances above saturation, rETR declined and this reduction was greater for the coral tissue spectrum treatment, as compared to the halogen lamp spectrum treatment. When incorporating photosynthetically usable radiation (PUR) into our calculations of electron transport, we found that this proxy for absolute electron transport rates (ETR; φ×PUR) showed a steeper rise for the coral tissue spectrum (α = 0.35±0.01 SE) compared to the halogen lamp spectrum (α = 0.26±0.003 SE) (Table 1; 2-tailed *t* test, t(17.8) = 4, p<0.001), while above saturation, ETR did not differ between the spectral treatments (Fig. 4B).

The relationship of O_2_ evolution and ETR (Fig. 4C) was linear for irradiance levels <400 µmol photons m^−2^ s^−1^; however, ETR declined relative to O_2_ evolution under higher irradiance (compare Fig. 3D and 4B). Calculations of NPQ showed a rapid increase at photon irradiances>250 µmol photons m^−2^ s^−1^, with peak values at the highest irradiance level (∼1000 µmol photons m^−2^ s^−1^) of 3.7 (±0.25 SE) for the halogen lamp spectrum and 2.8 (±0.52 SE) for the coral tissue spectrum (Fig. 4D).

## Discussion

We used scalar irradiance microsensors in combination with a novel programmable light source to study *Symbiodinium* photobiology under different defined spectral regimes at identical PAR levels. Use of the red-weighted halogen lamp illumination induced an intriguing physiological response, whereby gross extracellular O_2_ production was unchanged, the PSII photochemical efficiency (and hence ETR) decreased, while there was a trend towards enhanced LEDR.

Spectral quality of incident radiation has previously been found to affect O_2_ evolution rates in corals when illuminated with narrow bandwidth blue and red light [17], [49]. However, in our study, the reconstructed light spectra were broadband and smooth over the PAR region (Fig. 1) and under such more gradual spectral shifts between treatments, O_2_ evolution was not affected (Fig. 3D). Therefore, our observations suggest that the spectral quality of a conventional halogen light source does not significantly distort extracellular O_2_ evolution based estimates of gross photosynthesis rates compared to the natural *in hospite* light spectrum that was tested here (i.e. *Favites abdita* polyp in shallow water; Fig. 1A). The spectral light field that *Symbiodinium* receives within the tissue is variable and is modulated through tissue type and thickness [19], [21] and the quality of the incident irradiance, and thus water depth [27]. It will be interesting in the future to test how shifts in light quality on both macro- and microscale act together in affecting *Symbiodinium* photobiology.

Our rETR rates suggest that the blue-weighted excitation spectrum is most efficient at downregulating PSII activity (Fig. 4A). However, rETR is simply derived from the quantum yield of PSII and the photon irradiance integrated over PAR and thus does not account for the spectrally dependent absorption by *Symbiodinium* (Fig. 1A). Incorporation of the absorption spectrum of *Symbiodinium* yields an estimate of PUR (Fig. 1B) and an improved approximation of electron transport rates [50]–[51]. Using this proxy for ETR led to equal rates of electron transport for the red and blue weighted excitation spectrum under excess irradiance, which matched well with the observed measures of O_2_ evolution (compare Fig. 3D and 4B). However, for light-limiting conditions, estimated absolute electron transport rates and α were enhanced for the blue-weighted spectrum, although O_2_ evolution was not affected by the spectral treatments (Figs. 3D and 4B, Table 1). We suggest that this mismatch is likely caused by a combination of factors of which i) the presence of alternative electron pathways that serve as electron sinks without O_2_ evolution [52], and ii) spectrally-dependent conversion of absorbed light energy to chemically stored energy [4], [19] are most important. The functional absorption cross-section of PSII (i.e. the amount of light absorbed for PSII photochemistry) has recently been found to be higher in the blue than in the red part of PAR [19], consistent with the enhanced ETR measured under the blue-weighted spectrum in this study (Fig. 4B). At present, an improved understanding of the optical properties of *Symbiodinium* is clearly needed to better estimate light absorption by PSII, i.e., the PSII absorption cross section and consequently absolute electron transport rates [19].

LEDR strongly increased under enhanced irradiance levels (Fig. 3A and C). Under excess irradiance (>300 µmol photons m^−2^ s^−1^), it appeared that the LEDR rates of the red-weighted halogen lamp spectrum were moderately enhanced over the blue-weighted coral tissue spectrum (Fig. 3C). Although these are the first measurements of LEDR under different broadband spectra in *Symbiodinium*, they suggest that LEDR could be affected by the spectral composition of incident irradiance.

*Symbiodinium* use one or more O_2_ consuming alternative electron pathways to dissipate excess energy under high irradiance including cyclic electron transport around PSI [53], the Mehler reaction [54]–[56] and photorespiration [57]. Although it is not known whether O_2_ consuming pathways in *Symbiodinium* are enhanced under red light illumination, orange-red light (600–700 nm) is preferentially absorbed by PSI [15]; this would indicate that the enhanced O_2_ consumption for the red-weighted spectrum (Fig. 3C) could reflect enhanced PSI activity via Mehler [55] and/or PSI cyclic flow [53].

Decreased rETR at steady O_2_ evolution rates for both spectral treatments (Fig. 3C and 4A) further suggested *Symbiodinium* employed energy dissipation mechanisms in addition to the enhanced O_2_ consuming pathways. NPQ certainly played a role in dissipating excess energy in our study, as evidenced by the rapid increase in NPQ at irradiances above 300 µmol photons m^−2^ s^−1^ (Fig. 4D). However, NPQ cannot explain the high O_2_ evolution rates (Fig. 3D) at lowered rETR (Fig. 4A). Such mismatch has been reported previously [24], [58] and is likely the result of cyclic electron flow around PSII [24]. PSII-specific pathways would act to increase, or at least maintain, the PSII efficiency and ETR, but have so far not been described for *Symbiodinium* despite evidence for PQ pool reduction by mechanisms other than linear electron transport [59]. This latter point clearly indicates the need for more detailed knowledge of the “true” gross O_2_ production and hence the light-dependent O_2_ consumption rates and pathways, as well as accurate and absolute ETRs for PSII, to fully elucidate the complex nature of photochemical energy utilization observed here.

If LEDR is generally enhanced under red-weighted excitation for *Symbiodinium*, then this might be important for shallow water corals, which are subject to high quantities of red light [50]. Under supra-optimal irradiance regimes O_2_ concentrations in the coral tissue can be very high [29], [60] which can induce oxidative stress through the buildup of harmful O_2_ radicals [61]–[62]. High rates of O_2_ consumption activated by red light illumination (Fig. 3C) might therefore protect shallow water corals from oxidative stress. This aspect of wavelength dependent O_2_ consumption should certainly be studied in the future.

Corals are subject to different spectral qualities not only on larger macroscales but also on microscales. This leads to a coral-specific landscape of different light microenvironments for resident zooxanthellae. We found that extracellular O_2_ production of *Symbiodinium* is not affected by moderate spectral shifts between the *in situ* coral tissue spectrum and a conventional halogen lamp spectrum, while light use efficiency, as estimated through variable Chl *a* fluorescence, does differ. We suggest that this mismatch is likely related to light-dependent O_2_ consuming pathways. Our novel approach of using light microsensors and a programmable light engine to reconstruct and compare different spectral light regimes provides a framework to evaluate in detail how coral tissue optical properties influence metabolic and photophysiological functioning of symbiotic algae in different coral hosts and across different water bodies.

## Supporting Information

Figure S1 **Spectral distribution of scalar irradiance in % of the incident downwelling irradiance.** Measurements were performed 100 µm deep inside the polyp tissue of the coral *Favites abdita*.
(TIF)

File S1 **This file contains all displayed data.**
(XLSX)
