# Supplementary figures and images for: Spectral Effects on Symbiodinium Photobiology Studied with a Programmable Light Engine
Source: PLoS One. 2014 Nov 12;9(11):e112809. doi: 10.1371/journal.pone.0112809 (PMC4229233; doi:10.1371/journal.pone.0112809)

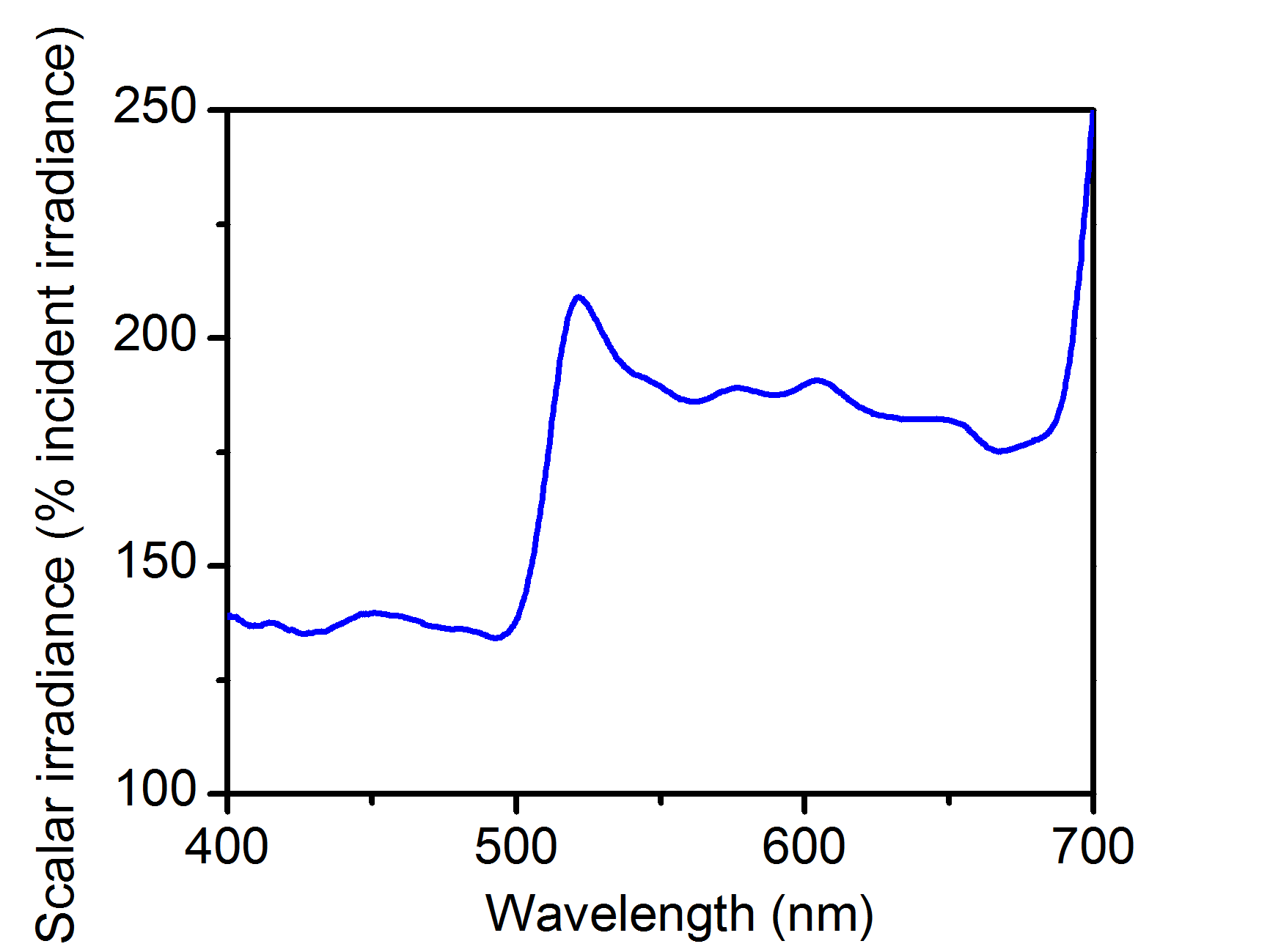

Supplement: Figure S1 — Spectral distribution of scalar irradiance in % of the incident downwelling irradiance. Measurements were performed 100 µm deep inside the polyp tissue of the coral Favites abdita. (TIF) [file pone.0112809.s001.tif]
